# Advances in experimental phasing

**DOI:** 10.1107/S2059798316003375

**Published:** 2016-03-01

**Authors:** Airlie J. McCoy, Thomas Schneider

**Affiliations:** a Department of Haematology, University of Cambridge, Wellcome Trust/MRC Building, Hills Road, Cambridge CB2 0XY, England; b European Molecular Biology Laboratory, Hamburg Unit, c/o DESY, Notkestrasse 85, 22607 Hamburg, Germany

**Keywords:** CCP4 Study Weekend, experimental phasing

## Abstract

An introduction to the 2015 CCP4 Study Weekend on Advances in Experimental Phasing.

This issue publishes the proceedings of the 2015 CCP4 Study Weekend on *Advances in Experimental Phasing*, held on 8–9 January 2015 at the East Midlands Conference Centre of the University of Nottingham, UK.

Crystallographic phasing using experimental approaches remains an important, and often the only successful, route for solving crystal structures, especially for structures with novel folds. These are often large and complex assemblies diffracting to limited resolution, but also those likely to provide the most novel biological insights. Our speakers were invited to cover both practical aspects, such as sample preparation and data collection strategies, and computational aspects, relating to the analysis and use of diffraction data from derivatized or anomalously scattering crystals. A session on radiation damage was included to remind experimentalists of the unavoidable side effects of using X-rays for diffraction data collection, while a final session on evolving methods provided a forum for ideas for (perhaps) solving the phase problem employing unconventional approaches.

The meeting began with a lecture by Neil Isaacs (University of Glasgow) sharing his personal view of the history of crystallographic phasing from the structure determination of zincblende, solved with the logic of constructive and destructive interference alone, *via* the heroic early macromolecular structure determinations by multiple isomorphous replacement, all the way to the routine exploitation of weak anomalous signals for the phasing of macromolecular crystals structures. Janet Smith (University of Michigan) then described the foundations of anomalous phasing demonstrating its current capabilities by way of a *tour-de-force* of phasing using raw data from many crystals.

To demonstrate the power of experimental phasing methods, the second session, after an introduction to the practicalities of preparing derivatized crystals by Liz Carpenter (University of Oxford), comprised three recent case studies of challenging structure determinations; cluster-compound based phasing of the multi-subunit membrane protein bestrophin-1 by Veronica Dickson (University of Cambridge); the determination of the COP9 signalosome structure by Richard Bunker (Friedrich Miescher Institute Basel); and the use of artificially introduced anomalous markers for the validation of the structure of the AP2 clathrin adapter by Bernard Kelly (University of Cambridge).

The scientific program of the first day of the Study Weekend was concluded with a session on the computational side of experimental phasing methodologies. Paula Salgado (Newcastle University) gave an overview of the different ways of exploiting dispersive and/or anomalous differences measured on one or several potentially non-isomorphous crystals. Tom Terwilliger (Los Alamos National Laboratory) introduced a new metric for assessing the potential of SAD data to lead to a successful structure solution. Randy Read (University of Cambridge) then discussed a log-likelihood-gain based framework for an improved accounting of measurement errors and uncertainties by means of a formulation in terms of diffraction intensities instead of the structure-factor amplitudes. Garib Murshudov (MRC Laboratory of Molecular Biology) described how experimental phases can be used as additional observations in structure refinement. Having last been discussed at a CCP4 Study Weekend in 2009 (http://journals.iucr.org/d/issues/2010/04/00/), radiation damage as a major problem in experimental phasing was highlighted in the first session of the second day. Robin Owen (Diamond Light Source) dissected the effect of radiation- and/or derivatization induced structural changes by carefully inspecting the effects on individual reflections. How radiation damage, deliberately induced by UV irradiation, can be harnessed, through optimized data collection schemes was demonstrated by Max Nanao (EMBL Grenoble). The design and the implementation of interleaved data collection protocols for the minimization of systematic errors due to radiation damage effects was described by Gerard Bricogne (Global Phasing Ltd). The use of data from multiple crystals as a means of dealing with limited life-time of individual crystals was then discussed by Gwyndaf Evans (Diamond Light Source).

The next session featured representatives from several synchrotrons advocating the modern experimental approaches available. Michele Cianci (EMBL Hamburg) showed how micro-focused low-energy X-ray beams can be used to extract optimum anomalous data from small crystals. The advantages of multi-circle goniometry in terms of increasing multiplicity and correcting for systematic errors was discussed by Andy Thompson (Synchrotron SOLEIL). Daniele de Sanctis (ESRF) described experimental options for achieving optimum data in different scenarios, while Vincent Olieric (Swiss Light Source) described generally applicable strategies for native SAD phasing exploiting anomalous signals from sulfur and or phosphorus. Finally, Armin Wagner (Diamond Light Source) described progress and first results from the new I23 in vacuum beamline at the Diamond synchrotron.

The final session of the Study Weekend concerned evolving methods. The successful use of molecular replacement for boot-strapping heavy-atom localizations in large macromolecular complexes in a parallelized computer implementation was described by Bjorn Panyella Pedersen (Aarhus University). The potential use of introducing differences in dispersive and anomalous signals by X-FEL radiation and the subsequent exploitation of these differences for phase determination was explained by Sang-Kil Son (CFEL-DESY, Hamburg). Finally, two methods of iteratively generating improved phase sets by were presented. Monarin Uervirojnangkoorn (Stanford University) employed genetic algorithms for the production of updated phase sets. Julien Jorda (UCLA) demonstrated the use of human perception for the scoring of updated electron densities with crowdsourcing software, which required audience participation.

We would like to express our gratitude to the CCP4 staff, in particular Karen McIntyre, for their invaluable help with the practical aspects of the 2015 Study Weekend. We thank all our speakers for their careful preparation and enthusiastic presentation of their talks. We hope that their papers will inspire young and seasoned crystallographers alike.

